# Supplementary material for: Reconciling Mining with the Conservation of Cave Biodiversity: A Quantitative Baseline to Help Establish Conservation Priorities
Source: PLoS One. 2016 Dec 20;11(12):e0168348. doi: 10.1371/journal.pone.0168348 (PMC5173368; doi:10.1371/journal.pone.0168348)
Supplement: S1 Dataset — (ZIP) [file pone.0168348.s002.zip › Taxa/Serra Norte/SN_2013/N4E/Lista N4E-09.pdf]

CAVIDADE N4E-0009

Classe	Ordem	Fam/Outros	Gên/Outros	Espécie	S-U	U-S	Zon.
Arachnida	Acari	Opilioacaridae		sp.1	X		E
Arachnida	Acari	Prostigmata		sp.	X		E
Arachnida	Acari	Rhagidiidae		sp.5		X	E
Arachnida	Acari	Trombidiidae	Microtrombidiinae	sp.	X		E
Arachnida	Araneae	Ctenidae		jovem	1		E
Arachnida	Araneae	Drymusidae		jovem		X	E
Arachnida	Araneae	Filistatidae		sp.1	X	X	E
Arachnida	Araneae	Linyphiidae		jovem	X		E
Arachnida	Araneae	Ochyroceratidae	*Speocera*	sp.1		X	E
Arachnida	Araneae	Pholcidae	*Mesabolivar*	sp.1		X	E
Arachnida	Araneae	Salticidae		sp.			E
Arachnida	Araneae	Scytodidae	*Scytodes*	sp.			E
Arachnida	Araneae	Theraphosidae	aff. *Holothele*	sp.1	X	2	E
Arachnida	Araneae	Theridiosomatidae	*Plato*	sp.1	X	X	E
Arachnida	Opiliones	Escadabiidae		sp.	X		E
Arachnida	Opiliones	Manaosbiidae	*Saramacia*	sp.1		1	E
Arachnida	Pseudoscorpiones	Chthoniidae	*Pseudochthonius*	sp.1		X	E
Chilopoda	Scolopendromorpha	Scolopocryptopidae	*Newportia*	*ernsti*	X		E
Chilopoda	Scutigeromorpha	Pselliodidae	*Sphendononema*	*guildingii*	X		E
Entognatha	Collembola	Entomobryidae		sp.4		X	E
Entognatha	Collembola	Paronellidae		sp.1		X	E
Entognatha	Collembola	Paronellidae		sp.7		X	E
Entognatha	Collembola			sp.	X		E
Entognatha	Diplura	Campodeidae		sp.1	X	X	E
Entognatha	Diplura	Projapygidae		sp.1	X		E
Insecta	Blattodea			jovem	X	1	E
Insecta	Coleoptera	Carabidae		jovem	X		E
Insecta	Coleoptera			jovem		X	E
Insecta	Diptera	Chironomidae		sp.	X		E
Insecta	Diptera	Culicidae		sp.	X		E
Insecta	Diptera	Psychodidae	Phlebotominae	sp.		X	E
Insecta	Diptera	Tipulidae	Tipulinae	sp.		X	E
Insecta	Heteroptera	Cydnidae		jovem		X	E
Insecta	Heteroptera	Reduviidae	Emesinae	sp.	X		E
Insecta	Heteroptera	Reduviidae	*Zelurus*	sp.	X	1	E
Insecta	Homoptera	Cixiidae		sp.6		X	E
Insecta	Hymenoptera	Bethylidae		sp.	X		E
Insecta	Hymenoptera	Formicidae	*Camponotus*	*crassus*	X		E
Insecta	Hymenoptera	Formicidae	*Dolichoderus*	aff. *bispinosus*		X	E
Insecta	Hymenoptera	Formicidae	*Pachycondyla*	sp.1		X	E
Insecta	Hymenoptera	Formicidae	*Pachycondyla*	sp.2		X	E
Insecta	Hymenoptera	Formicidae	*Pyramica*	*elongata*	X		E
Insecta	Isoptera	Termitidae	*Embiratermes*	sp.		X	E
Insecta	Isoptera	Termitidae	*Nasutitermes*	sp.		X	E
Insecta	Isoptera	Termitidae		sp.	X		E
Insecta	Lepidoptera	Tineoidea		sp.1	X	X	E
Insecta	Orthoptera	Phalangopsidae	*Paraclodes*	jovem		1	E
Insecta	Psocoptera			jovem		X	E
Malacostraca	Isopoda	Armadillidae		sp.1		X	E
Malacostraca	Isopoda	Dubioniscidae		sp.1		X	E
Malacostraca	Isopoda	Philosciidae		sp.1		X	E
Pauropoda	Tetramerocerata			sp.		X	E
Turbellaria	Tricladida			sp.3		1	E
Amphibia	Anura			sp.		3	E
